# Supplementary figures and images for: Instruments used to measure knowledge and attitudes of healthcare professionals towards antibiotic use for the treatment of urinary tract infections: A systematic review
Source: PLoS One. 2022 May 24;17(5):e0267305. doi: 10.1371/journal.pone.0267305 (PMC9129047; doi:10.1371/journal.pone.0267305)

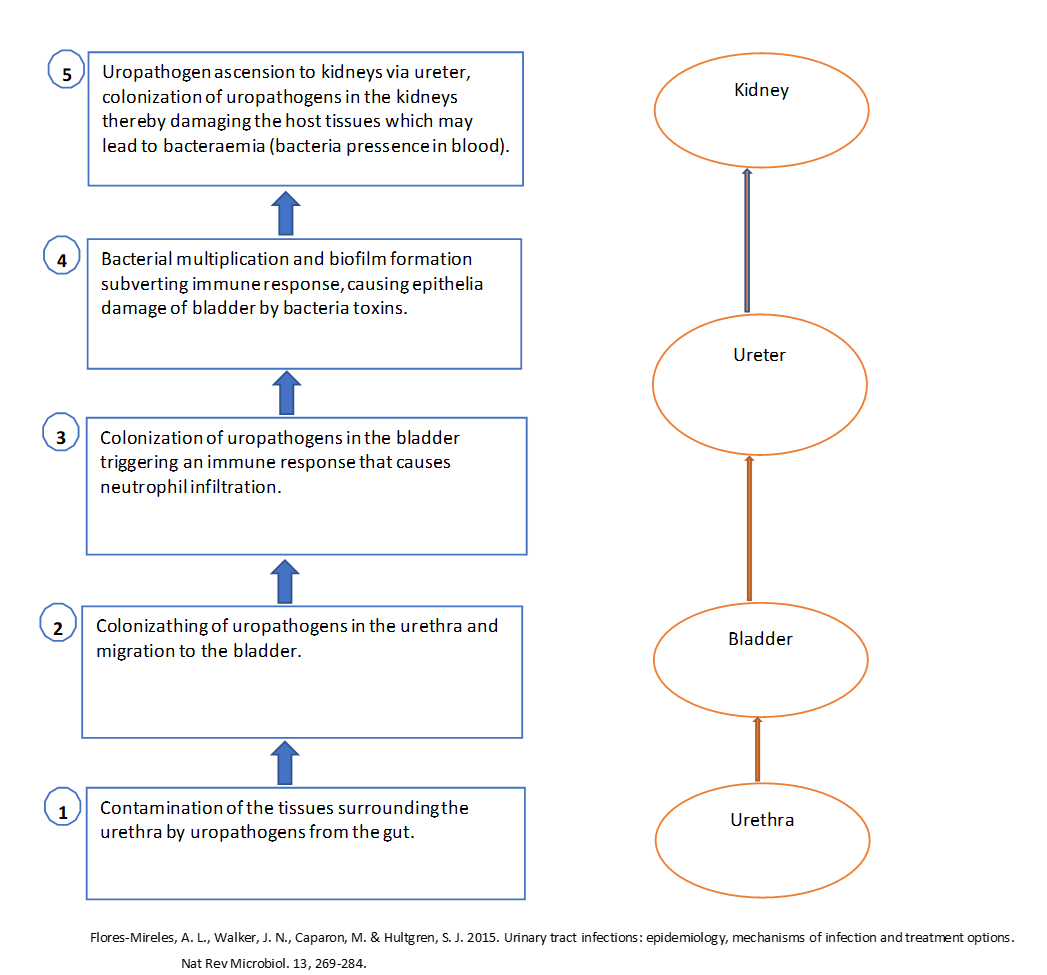

Supplement: S1 Fig — (TIF) [file pone.0267305.s001.tif]

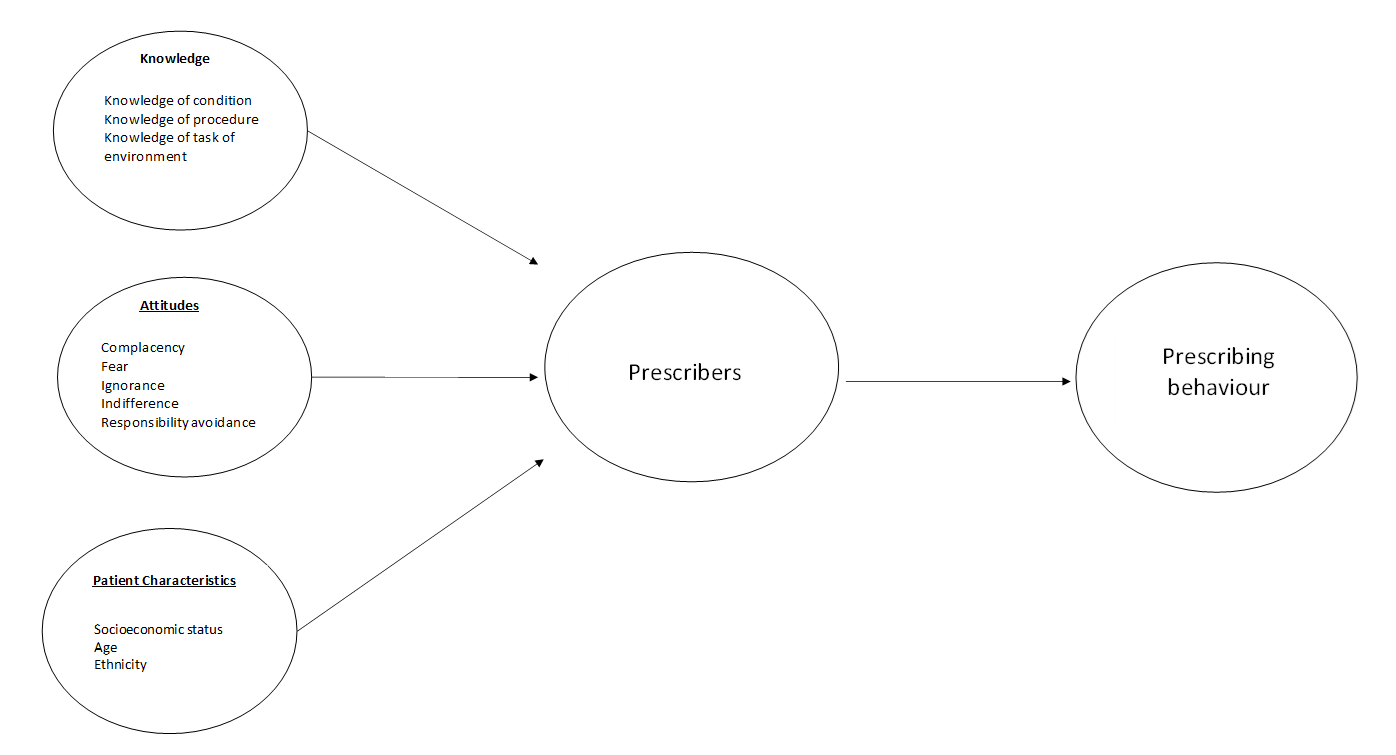

Supplement: S2 Fig — (TIF) [file pone.0267305.s002.tif]

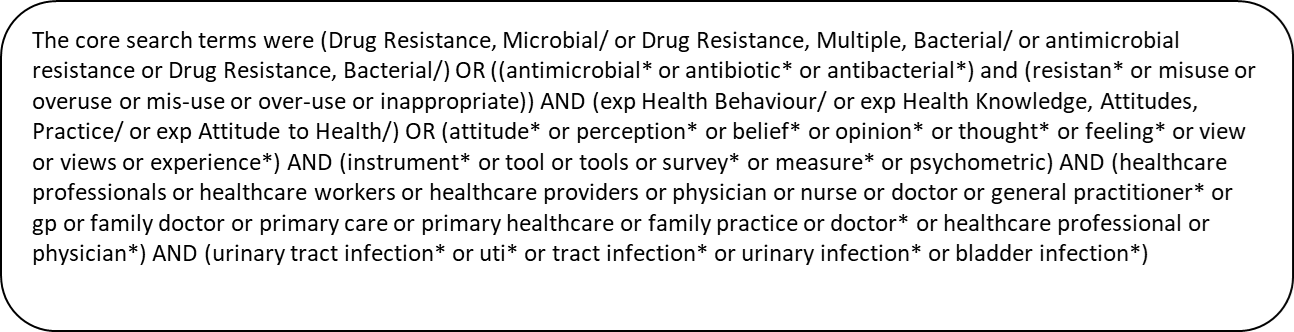

Supplement: S4 Fig — (TIF) [file pone.0267305.s004.tif]
