# Supplementary material for: Instruments used to measure knowledge and attitudes of healthcare professionals towards antibiotic use for the treatment of urinary tract infections: A systematic review
Source: PLoS One. 2022 May 24;17(5):e0267305. doi: 10.1371/journal.pone.0267305 (PMC9129047; doi:10.1371/journal.pone.0267305)
Supplement: S1 Table — (PDF) [file pone.0267305.s005.pdf]

Inclusion Criteria	Exclusion Criteria
Instrument measuring knowledge and attitudes of individual healthcare professionals including pharmacists, Nurses and Doctors towards the use of antibiotics to treat Urinary Tract Infections.	Surveys focusing on antibiotic use or ABR (antibiotic resistance) with only one question on antibiotic use and or ABR in the context of urinary tract infections.
Focus of study needs to be on the use antibiotics to treat Urinary Tract Infections by healthcare professionals with a follow-up on UTI-specific knowledge, attitudes and behaviour.	Surveys in the context of Urinary Tract Infection but focus on Students.
If the study looks at prescribing decisions, it must also look at KA (could be reasons for prescribing decision) and/or it must evaluate the appropriateness of those prescribing decisions and present findings on that assessment.	Surveys in the context of Urinary Tract Infection but focus on specific UTI (Urinary Tract Infections) causing bacteria.
Studies assessing all non-catheterised UTIs/ and or related infections that may require the use of antibiotics in the treatment pathway such as Lower UTI (Cystitis), Upper UTI (acute Pyelonephritis), Recurrent UTI and Asymptomatic Bacteriuria.	Surveys which assess knowledge and attitudes but regarding catheter associated urinary tract infections.
Study must evaluate one or more of the utility criteria (i.e., validity, reliability, acceptability, feasibility, and educational impact).	Surveys which focus on Antimicrobial Stewardship (AMS), use of one specific drug only, factors leading to AB misuse/ABR instead of knowledge and attitudes towards the use of antibiotics to treat UTIs.
Studies that measure an attitude (as opposed to asking about what you attitude is)	Assess KAB in terms of prophylaxis for UTIs.
Studies focusing on qualitative methods to get a quantitative measure.	Studies that assessed choice of antibiotic only and not about whether to give antibiotics (treatment choice)
Any study designs.	Assessed need for evidence on UTI treatment than treatment decision.
	Studies that assessed General and non-specific attitude questions in the context of UTIs
	Studies which have no evaluation of a utility criterion (i.e., validity, reliability, acceptability, feasibility, and educational impact)
	Surveys that assessed knowledge and attitudes using a consensus panel
	No study designs.
	Non- English articles.
