# Supplementary material for: Instruments used to measure knowledge and attitudes of healthcare professionals towards antibiotic use for the treatment of urinary tract infections: A systematic review
Source: PLoS One. 2022 May 24;17(5):e0267305. doi: 10.1371/journal.pone.0267305 (PMC9129047; doi:10.1371/journal.pone.0267305)
Supplement: S4 Table — (PDF) [file pone.0267305.s008.pdf]

S4 Table Quality Criteria for Measurement Properties (Terwee et al. 2007

Property	Rating	Quality Criteria
**Validity**		
Content validity	(+)	The target population considers all items in the questionnaire to be relevant AND considers the questionnaire to be complete
	?	No target population involvement
	(−)	The target population considers all items in the questionnaire to be irrelevant OR considers the questionnaire to be incomplete
Construct validity		
Structural validity	(+)	Factors should explain at least 50 % of the variance
	?	Explained variance not mentioned
	(−)	Factors explain <50 % of the variance
Hypothesis testing	(+)	Correlation with an instrument measuring the same construct ≥50 % OR atleast 75 % of the results is in accordance with the hypotheses) AND correlation with related constructs is higher than with unrelated constructs
	?	Solely correlations determined with unrelated constructs
	(−)	Correlation with an instrument measuring the same construct <50 % OR <75 % of the results is in accordance with the hypotheses OR correlation with related constructs is lower than with unrelated constructs
**Reliability**		
Internal consistency	(+)	(Sub)scale unidimensional AND Cronbach’s alpha(s) ≥0.70
	?	Dimensionality not known OR Cronbach’s alpha not determined
	(−)	(Sub)scale not unidimensional OR Cronbach’s alpha(s) <0.70
Measuremnt error	(+)	MIC > SDC OR MIC outside the LOA
	?	MIC not defined
	(−)	MIC ≤ SDC OR MIC equals or inside LOA
Reliability	(+)	ICC/weighted Kappa ≥0.70 OR Pearson’s r ≥ 0.80
	?	Neither ICC/weighted Kappa, nor Pearson’s r determined
	(−)	ICC/weighted Kappa <0.70 OR Pearson’s r < 0.80

+ positive, − negative, ? indeterminate, AUC area under the curve, MIC minimal important change, ICC intraclass correlation, SDC smallest detectable change, LOA limits of agreement.

Terwee, C., Bot, S., Boer, M., Van Der Windt, D., Knol, D., Dekker, J., Bouter, L. & De Vet, H. 2007. Quality criteria were proposed for measurement properties of health status questionnaires. Journal of clinical epidemiology, 60, 34-42.
