# Supplementary material for: Instruments used to measure knowledge and attitudes of healthcare professionals towards antibiotic use for the treatment of urinary tract infections: A systematic review
Source: PLoS One. 2022 May 24;17(5):e0267305. doi: 10.1371/journal.pone.0267305 (PMC9129047; doi:10.1371/journal.pone.0267305)
Supplement: S5 Table — (PDF) [file pone.0267305.s009.pdf]

3 x 3 Table of decisions

R1 + R2	Y	M	N	Total
**Y**	8	0	1	9
**M**	3	11	5	19
**N**	2	10	2389	2401
**Total**	13	21	2395	2429

Key: R1 = reviewer 1, R2 = reviewer 2, Y=Yes, M=maybe and N=No

Number of observed agreements: 2408 (99.14% of the observations)

Number of agreements expected by chance: 2367.6 (97.47% of the observations)

Kappa= 0.658

SE of kappa = 0.067

95% confidence interval: From 0.527 to 0.789
