# Supplementary material for: Instruments used to measure knowledge and attitudes of healthcare professionals towards antibiotic use for the treatment of urinary tract infections: A systematic review
Source: PLoS One. 2022 May 24;17(5):e0267305. doi: 10.1371/journal.pone.0267305 (PMC9129047; doi:10.1371/journal.pone.0267305)
Supplement: S6 Table — (PDF) [file pone.0267305.s010.pdf]

	Knowledge			Attitudes								
Study	Scientific rationale	Knowledge of condition	Procedural knowledge	Task of environment	Patient characteristics	Complacency	Fear	Ignorance	Indifference	Responsibility of others	Confidence	Patient characteristics
[41]	knowledge of first- and second-line antibiotic use	knowledge of UTI symptoms : haematuria, renal colic , micturition urgency, foul smelling urine and turbid urine	patient pressure	implementation of regulations enforcement: adherence to pharmaceutical law	knowledge of patients socioeconomic status (average cost of medication) and age	cost reduction for patient						
		knowledge of the relation of dysuria with sexual activity			patients previous medication							
		knowledge of duration of UTI symptoms										
[42]	knowledge of antimicrobial route, dose and frequency	knowledge of UTI symptoms of urinary frequency, dysuria and haematuria and signs of persistent febrility (38C) with mildly tender bladder	knowledge and interpretation of dipstick test (nitrite and leucocyte) and biochemistry tests (erythrocyte sedimentation rate (ESR) and CRP levels > normal)	influence on decision to prescribe reference to external resources	patient medical history, marital status and age					admission of patient into hospital		
[32]	awareness of increasing resistant organisms	Knowledge of UTI symptoms : dysuria and frequent urination		influence of regulatory authority	health of patient	complacency towards patients obtaining antibiotics from another pharmacy if not dispensed	fear of losing patients if they do not dispense them with an antibiotic			non-malfeasance principle is not violated: encouraging patients to consult with physicians	education about importance and completion of antibiotic course	
	awareness of the contribution of dispensing antibiotics without prescription to AMR			patient pressure	patients age and childbearing potential (pregnancy status)		fear that refusing DAwP will negatively affect sales and profits				confidence in patient presentation of symptoms and signs through interviewing without need prescription	

	fulfilment of patient care through counselling about the importance of treatment adherence and appropriate antibiotic use (discussing allergies, dosing, need to complete the full course)									
[33]	awareness of local AMR patterns to decide on treatment	knowledge of symptoms and diagnosis of patients with uncomplicated UTI	procedural knowledge of when to conduct UTI test	influence of microbiological laboratory and updates			fear of treatment failure associated with resistance	habitual use of antibiotics		familiarity with patients
		knowledge of when to give direct or delayed treatment		influence from healthcare system structure						
		knowledge of how to treat recurrent UTI symptoms								
[38]		knowledge of dosing, duration of treatment and drug allergy for each type of UTI	knowledge of interpretation of urine dip results for nitrites, leucocytes and blood tests (white cell count, C-reactive protein and renal function within the normal range)	influence of external resources on antibiotic prescribing choices e.g previous hospital's practice, advice/directive from senior doctors, local guidelines, medical school teaching, national guidelines, observed practice in current hospital, other guidelines, post-qualification teaching, research data	patients medical history (e.g previous UTIs, hypertension, history of resistant pathogens), allergies and pregnancy status					

		knowledge of various presentations of uncomplicated UTI (cystitis), pyelonephritis, cellulitis		
		knowledge of uncomplicated UTI symptoms such as urinary frequency and dysuria for 24 hours, haemodynamically stable, low-grade pyrexia of 37.6 °C on presentation and signs such as mild suprapubic tenderness but no clinical evidence of pyelonephritis		
		knowledge of pyelonephritis symptoms such as fever, rigors, and left-sided flank pain with uncomplicated UTIs and signs such as blood pressure (BP) is 130/80, heart rate is 110 beats per minute (bpm) and temperature is 38.5°C,soft abdomen.		
[36]	knowledge of evidence-based practice	knowledge of the treatment and management of asymptomatic bacteriuria	influence of scientific evidence/ evidence-based guidelines	complacency towards guidelines
		knowledge of suspected UTI conditions that require antibiotic treatment		
		knowledge of the treatment and management of asymptomatic bacteriuria	influence of scientific evidence/ evidence-based guidelines	

		knowledge of suspected UTI conditions that require antibiotic treatment							
[37]	awareness of increasing resistant organisms (Gram negative)	awareness of UTI related symptoms such as foul-smelling urine, bacteria in the urine, dysuria and urinary incontinence and signs such as new costovertebral tenderness	procedural knowledge of monitoring patients with history of UTI, fever and no other complaints: monitor vital signs more frequently, recommend antibiotics, encourage fluids	educational activities - online videos, written materials, presentations, and activities	changes patient functional status, recent falls, new onset confusion and history of UTI	perception that antibiotics contributes to high quality care	fear of side effects : diarrhoea, rash, allergic reactions, medication interactions, c.difficile, antibiotic resistance		in ability to recognise symptoms of UTI
				family concern about a possible infection					
	awareness of associations between antibiotics and several side effects (such as rash, allergic reactions and antibiotic resistance)	awareness of UTI classifications and their symptom representations which necessitate antibiotic prescribing		the role of nurses serving as a liaison between prescribers and family (their position to explain why antibiotics are not being used and their adverse effects)					the harm and benefits of antibiotics
		treatment ambiguity of patients who are asymptomatic with antibiotics							ability to explain to patient/family why antibiotics are unnecessary

[34]	knowledge of local area resistance patterns		grade or experience of physician i.e. appropriate prescribing in more senior doctors	patient cohorts with need of isolation		side effects due to overuse in human medicine		insufficient hand disinfection and hygiene standards among medical staff	confidence regarding dosage, frequency, and duration of antibiotic treatment among urologists as well as interpreting antibiograms	
	knowledge of local area resistance patterns		Therapeutic guidelines			overuse of broad-spectrum antibiotics			confidence regarding local resistance patterns among urologists	
	knowledge of antimicrobial stewardships		national AMR programs						confidence in correct decision regarding the indication of intravenous or oral application of antibiotics	
	knowledge of broad-spectrum antibiotics that result in increased resistance pattern		training courses regarding multi-drug resistance or antibiotic prescribing						the correct interpretation of microbiological reports	
	knowledge of amount of local antibiotic prescribing		hygiene measures and hygiene standards in the hospital						indication of antibiotic therapy	
	knowledge of indications of MRSA screening		the current rules for hand disinfection							

knowledge of indications of MDRGN-screening	the possibilities of success monitoring of sufficient hygiene measures and hygiene standards
knowledge of mixing and cloning of antibiotic treatment regimes	responsibility of identifying notifiable infectious diseases
knowledge of shortened or extended administration of antibiotics	insufficient surveillance measure on the rational use of antibiotics
	insufficient knowledge and guideline adherence regarding the rational use of antibiotics
	insufficient advance training and no mandatory advanced training for medical stuff
	internal hospital guidelines and hospital standards
	official national/international guidelines
	microbiological advice
	colleague advice
	lack of trained staff in hospitals and private practices
	lack of international and global strategies in fighting increasing antimicrobial resistance
	overuse or extended use of foreign bodies e.g foley catheters - potentially favouring infections
	insufficient research activity with pharmaceutical companies on novel potent antibiotics
	too much influence by pharmaceutical companies

[43]	knowledge of resistant and susceptible antibiotics to UTI pathogens	knowledge of symptom representation for the various types of UTIs (uncomplicated cystitis, recurrent UTI, immunosuppressed, no UTI, pyelonephritis, urethritis, no UTI.	knowledge of when to treat with an antibiotic and not send for culture and sensitivity testing	knowledge of evidence-based guidelines e.g., therapy for uncomplicated cystitis was considered to be trimethoprim/sulfamethoxazole, nitrofurantoin, Fosfomycin, or pivmecillinam	patients past UTI history and most recent culture (e.g., 3 months, 6 months, a year , none)		physician's confidence with use and interpretation of rapid UTI diagnostics
		knowledge of uncomplicated UTI symptoms e.g dysuria, urinary frequency and urgency, signs e.g normotensive, afebrile, no costovertebral angle (CVA) tenderness	knowledge of when to treat with an antibiotic and send for culture and sensitivity testing	knowledge of evidence-based guidelines e.g therapy for acute pyelonephritis was outlined as ciprofloxacin, trimethoprim/sulfamethoxazole, ceftriaxone, or an aminoglycoside.	patients comorbidities e.g immunosuppression from daily methotrexate use		
		knowledge of recurrent UTI symptoms e.g symptoms and signs of UTI but with most recent culture dysuria, urinary frequency and urgency, signs e.g normotensive, afebrile, no costovertebral angle (CVA) tenderness	knowledge of when to send urine for culture and sensitivity and postpone treatment pending results (delayed antibiotic prescribing)				

	knowledge of UTI symptoms that do not necessitate antibiotic treatment such as patient with recurrent UTI and immunosuppression from daily methotrexate with urinary frequency , urgency and pelvic pain symptoms and signs of normotensive, afebrile, non-specific back pain , CVA tenderness and recent culture of 3 months ago with detected *k.pneumoniae* but no bacteria detected	knowledge of when to perform a urine dipstick test and interpretation (leukocyte esterase>trace, nitrites>trace, blood > trace)
		knowledge of when to take a urine sample for culture and interpretation (i.e positive if the colony count was greater than or equal to 1000 CFU/mL (1000000 CFU/L)) of a single known pathogen.

[44]		knowledge of UTI symptoms that may or may not necessitate an antibiotic e.g dysuria, frequency, urgency, nocturia, postvoid urgency, suprapubic pressure, voids small amounts, flank discomfort, vaginal discharge, previous UTI	knowledge of when to perform a urine dipstick test and interpretation (leukocyte esterase>trace, nitrites>trace, blood > trace)		patients age and marital status
		knowledge of UTI signs that necessitate an antibiotic e.g suprapubic tenderness, CVA tenderness	knowledge of when to take a urine sample for culture and interpretation (i.e positive if the colony count was greater than or equal to 1000 CFU/mL (1000000 CFU/L) of a single known pathogen.		
		knowledge of UTI clinical characteristics that necessitate an antibiotic			
[40]		ambiguity in the definition of asymptomatic bacteriuria and asymptomatic bacteriuria			
		knowledge of symptoms in the presence of bacteriuria in an elderly individual that affect decision to prescribe new or increased dysuria or urgency			

	knowledge of symptoms in the presence of bacteriuria in an elderly individual that affect decision to prescribe new or increased incontinence	
	knowledge of symptoms in the presence of bacteriuria in an elderly individual that affect decision to prescribe decline in mental or functional status (a fall, loss of appetite, increased agitation)	
	knowledge of symptoms in the presence of bacteriuria in an elderly individual that affect decision to prescribe malodorous urine, cloudy urine, bloody urine, chills	

knowledge of conditions that require monitoring for symptomatic bacteriuria in elderly i.e difficult presenting symptoms (demented, immuno-compromised), recurrent UTIs and/or those receiving prophylactic UTI measures, diabetes, kidney function , many comorbidities (frail elderly), nursing home residents for infection control purpose only (not to treat bacteriuria)
knowledge of symptoms in the presence of bacteriuria in an elderly individual that affect decision to prescribe new or worsening pain - suprapubic, flank or costovertebral angle, temperature >37.9C or 100F or 1.5C (2.4F) above baseline
knowledge that antibiotic treatment for bacteriuria without symptoms is not recommended for the geriatric patient

		knowledge if the presence of pyuria necessitates antibiotic therapy										
[39]		knowledge of patient symptoms that may not always require treatment when assessed independently such as dysuria , increased frequency of micturition and other symptoms (vaginal symptoms , abdominal symptoms, back pain, haematuria, nocturia fever, urgency, p.vulvae infection)	procedural knowledge of obtaining a clean catch midstream urine sample for pathology services and interpreting previous/current results of urine analysis	patient pressure		knowledge of patient social class, probable psychological disorder, and menstrual problems	patient acquaintance					
						physicians knowledge of patients age, sex , marital status and occupation						
[35]		knowledge of appropriate antibiotic treatment for young pregnant women (26 years old , 24 weeks) who presents with burning pain on urination for one day								referral following no symptom improvement following three days	confidence in over-the-counter prescription of antibiotics by pharmacists including those for treating UTIs such as trimethoprim, nitrofurantoin	patients confidence in pharmacist advise for conditions that require antibiotic use

		knowledge of when to select another antibiotic after 3 days of no resolution of UTI symptoms		older respondents (pharmacists) more likely to refer patients to GPs and less likely to prescribe appropriately
		knowledge of when to increase dose of antibiotic after 3 days of no resolution of UTI symptoms		
		knowledge of when to consider alternative diagnosis and treat accordingly after 3 days of no resolution of UTI symptoms		
		knowledge of when to refer to another GP after 3 days of no resolution of UTI symptoms		
[45]		awareness of patient symptoms which necessitate antibiotic prescription	ordering and interpreting urine culture	
