# Supplementary material for: Instruments used to measure knowledge and attitudes of healthcare professionals towards antibiotic use for the treatment of urinary tract infections: A systematic review
Source: PLoS One. 2022 May 24;17(5):e0267305. doi: 10.1371/journal.pone.0267305 (PMC9129047; doi:10.1371/journal.pone.0267305)
Supplement: Supplementary file 9 [file pone.0267305.s006.odt]

S2 Table : Definition of study terms  

| Terminology | Definition |
| --- | --- |
| Peer-reviewed original literature | Peer-reviewed articles published in journals presenting novel data and or analysis |
| Knowledge and attitude Instrument | An instrument was considered to be a knowledge and attitude instrument if the instrument purported to assess knowledge and or attitudes |
| Psychometric properties | An instrument was considered to have had its validity, reliability, acceptability, feasibility and/or a potential for educational impact evaluated |
| Healthcare professionals | Practicing healthcare professionals including doctor or  nurse or pharmacists or Nurse or urologist |

Adapted from:  Carlson, M. A., Morris, S., Day, F., Dadich, A., Ryan, A., Fradgley, E. A. & Paul, C. 2021. Psychometric properties of leadership scales for health professionals: a systematic review. Implement Sci. 16, 85.
