# Supplementary material for: Instruments used to measure knowledge and attitudes of healthcare professionals towards antibiotic use for the treatment of urinary tract infections: A systematic review
Source: PLoS One. 2022 May 24;17(5):e0267305. doi: 10.1371/journal.pone.0267305 (PMC9129047; doi:10.1371/journal.pone.0267305)
Supplement: Attachment — Submitted filename: PONE-D-21-35085.docx [file pone.0267305.s011.docx]

The systematic review entitled “**Instruments used to measure knowledge and attitudes of healthcare professionals towards antibiotic use for the treatment of urinary tract infections: A systematic review**” which has highlighted the important aspects of the knowledge and attitudes of healthcare professionals towards antibiotic use for the treatment of urinary tract infections. The following are my comments for the **Authors**.

1. Overall, the present review if of great importance but the authors missed the main important aspect of KAP studies in relation to the healthcare professionals and that’s ***“Practices”*** of antibiotics for UTIs. As the along with the knowledge, attitude; practices are the prime factor in the KAP models studies and measurement. A reasonable explanation is needed.
2. The introduction must be started from the very basic definition of the UTIs and the cause of UTIs after that the epidemiology for of UTI may include.
3. The scale of measurement for the Psychometric approach must be clear in the methods section and how it was employed to the inclusion criteria. How was the PICO model fit in the inclusion criteria and which basis the studies were included, either on PICO or Psychometric measurement?
4. Are the included studies were finalized after the risk biased assessment? Is any scale was used for the risk bias assessment and the protocols at all.
5. The main evidence which has been drawn from the present review is not cleared. Very specific evidence must be mentioned that must represent the whole picture of the review. Such measuring scales and other factors must be controlled and the conclusion and the evidence from the review must be cleared that would be helpful for the readers and researchers in the field.
6. Recent references are needed to be included in the introduction and discussion parts.

Wish you all the best.

Thanks, and Best Regards.,
