# Supplementary material for: Instruments used to measure knowledge and attitudes of healthcare professionals towards antibiotic use for the treatment of urinary tract infections: A systematic review
Source: PLoS One. 2022 May 24;17(5):e0267305. doi: 10.1371/journal.pone.0267305 (PMC9129047; doi:10.1371/journal.pone.0267305)
Supplement: Attachment — Submitted filename: Response to Reviewers_03March22.docx [file pone.0267305.s012.docx]

In this revision letter we describe how we responded to the editor’s and reviewers’ comments. We found these comments incredibly useful and helpful for us to improve our manuscript. Where possible we state where changes were made in the manuscript (with page and line numbers). All authors have approved these changes.

**Editor comments:**

Editor 1 comment 1. Please ensure that your manuscript meets PLOS ONE's style requirements, including those for file naming. The PLOS ONE style templates can be found at 
<https://journals.plos.org/plosone/s/file?id=wjVg/PLOSOne_formatting_sample_main_body.pdf> and 
<https://journals.plos.org/plosone/s/file?id=ba62/PLOSOne_formatting_sample_title_authors_affiliations.pdf>

>>>Author Response to Editor 1 comment 1. Thank you for drawing our attention to the formatting samples. We have modified the text to include the new header structure throughout in Microsoft Word.

Editor 1 comment 2. Thank you for stating the following in the Acknowledgments Section of your manuscript:

“The current project was supported by the National Institute for Health Research (NIHR) Applied Research Centre (ARC) West Midlands, grant number NIHR200165. The views expressed are those of the author(s) and not necessarily those of the NIHR, ARC, or the Department of Health and Social Care. The funders had no role in the design of the study and collection, analysis, and interpretation of data and in writing the manuscript.”

Please note that funding information should not appear in other areas of your manuscript. We will only publish funding information present in the Funding Statement section of the online submission form.

Please remove any funding-related text from the manuscript and let us know how you would like to update your Funding Statement. Currently, your Funding Statement reads as follows:

“The current project was supported by the National Institute for Health Research (NIHR) Applied Research Centre (ARC) West Midlands, grant number NIHR200165. The views expressed are those of the author(s) and not necessarily those of the NIHR, ARC, or the Department of Health and Social Care. The funders had no role in the design of the study and collection, analysis, and interpretation of data and in writing the manuscript.”

Please include your amended statements within your cover letter; we will change the online submission form on your behalf.

>>>Author Response to Editor 1 comment 2. The funding statement has been removed from our manuscript. The funding is provided within our cover letter, and we thank the editorial team for changing the online submission form on our behalf.

**Reviewer 1 comments**

Reviewer #1: The systematic review entitled “Instruments used to measure knowledge and attitudes of healthcare professionals towards antibiotic use for the treatment of urinary tract infections: A systematic review” has highlighted the important aspects of the knowledge and attitudes of healthcare professionals towards antibiotic use for the treatment of urinary tract infections. The following are my comments for the Authors

>>>Author Response to reviewer 1 comment. Thank you. We are happy that this overall story come across in our manuscript.

Reviewer #1 comment 1. Overall, the present review if of great importance but the authors missed the main important aspect of KAP studies in relation to the healthcare professionals and that’s “Practices” of antibiotics for UTIs. As along with knowledge, attitude; practices are the prime factor in the KAP models studies and measurement. A reasonable explanation is needed.

>>>Author Response to reviewer 1 comment 1. We agree that the final determinant of antibiotic prescribing is the practices of healthcare professionals. The goal of the current study was not, however, to evaluate healthcare professionals’ knowledge, attitude, and practice but to report on the psychometric properties of instruments designed to measure healthcare professionals’ knowledge and attitudes. As we have elucidated, knowledge and attitudes are key drivers of prescribing practice. The rationale for the focus on the measurement of knowledge and attitudes in this study is that while practice can be measured using routine prescribing data or observation, knowledge and attitudes are constructs that are not directly observable, difficult to measure and must be well defined. Therefore, to measure knowledge and attitudes, it is important to ensure that the knowledge and attitudes items included in measurement instruments are developed based on sound theoretical frameworks and are psychometrically sound. The reason for the lack of inclusion of the “practice” component in this systematic review is now more clearly stated in the introduction, starting from line 118:

Although the constructs of Knowledge, Attitudes and Practice (KAP) are the key factors relating to measurement studies that utilise the WHO’s recommendations for developing KAP surveys (WHO 2008), the construct of practice was excluded from this review. This is because practice can be measured using routine prescribing data or physician observation in treatment of patients whereas knowledge and attitude are constructs that are not directly observable and are difficult to measure. To measure knowledge and attitude, as well as to understand their influence on practice, it is important to ensure that any measurement instrument is based on well-defined content areas of knowledge and attitude and has sound psychometric properties.

In the strength and limitations, we also now state, starting at line 486:

In line with the aims of this review, the “practice” component of the WHO’s knowledge, attitudes, and practice framework was excluded. Future studies could explore healthcare professionals’ practices – i.e., their actual prescribing of antibiotics for UTIs, and link practices with appropriate measures of knowledge and attitude.

Reviewer #1 comment 2.The introduction must be started from the very basic definition of the UTIs and the cause of UTIs after that the epidemiology for UTI may include. Line 64

>>>Author Response to reviewer 1 comment 2. We agree that our paper should provide more depth on the definition of UTIs and their causes. Thank you for your constructive comments that have surely improved the quality of the manuscript. We have now added this additional information starting at line 64. We have also provided an additional reference for readers who may wish to learn more about UTIs and included their pathogenesis in supplementary fig 1.

Heterogenous definitions of UTIs exist among researchers which are not always clear (Sartori et al., 2021). In broad terminology, UTIs can be defined as any infection involving any part of the urinary tract, namely urethra (urethritis), bladder (cystitis), ureters, or kidneys. UTIs are mainly caused by microorganisms such as Escherichia coli (E. coli), Staphylococcus saprophyticus, and Enterococcus faecalis (EAU, 2021). E. coli remains the predominant uropathogens accounting for 70-95% of cases (NICE, 2018). UTIs can be classified into five different categories as shown in Table 1. Research by Foxman (2014) and Flores-Mireles et al. (2015) provides additional information on risk factors of UTIs as well as their pathogenesis which is further explained in S1 Fig.

**Table 1: Classification of UTIs**

**Table 1: Classification of UTIs**

| Uncomplicated UTIs | | Acute, sporadic, or recurrent lower (uncomplicated cystitis) and /or upper (uncomplicated pyelonephritis) UTI, limited to non-pregnant women with no known relevant anatomical and functional abnormalities within the urinary tract or comorbidities. |
| --- | --- | --- |
| Complicated UTIs | | All UTIs which are not defined as uncomplicated. Meaning in a narrower sense UTIs in a patient with an increased chance of a complicated course: i.e., all men, pregnant women, patients with relevant anatomical or functional abnormalities of the urinary tract , indwelling urinary catheters, renal diseases, and/ or with other concomitant immunocompromising diseases such as diabetes. |
| Recurrent UTIs | | Recurrence of uncomplicated and/or complicated UTIs, with a frequency of at least three UTIs /year or two UTIs in the last six months. |
| Catheter-associated UTIs | | Catheter-associated urinary tract infection (CA-UTI) refers to UTIs occurring in a person whose urinary tract is currently catheterised or has had a catheter in place within 48 hours. |
| Urosepsis |  | Urosepsis is defined as life threatening organ dysfunction caused by a dysregulated host response to infection originating from the urinary tract and/or male genital organs. |

Reviewer #1 comment 3. The scale of measurement for the Psychometric approach must be clear in the methods section and how it was employed in the inclusion criteria. How was the PICO model fit in the inclusion criteria and on which basis the studies were included, either on PICO or Psychometric measurement?

>>>Author Response to reviewer 1 comment 3. We apologize that the psychometric approach was not clearly emphasized in the methods section. We agree that there is a lack of explanation in how the PICO model fits the inclusion criteria and admit this as an oversight on our part. As our review was non-interventional, we have now provided the search terms as was conducted without including any model. This is because we realize that the use of PICO model may have deviated attention to a review focusing on interventional studies which was not the case. This review sought to identify whether included instruments reported any psychometric properties. This is highlighted in the methods section, starting at lines 171 and 218 respectively:

To perform a highly inclusive search, the key search terms were refined in consultation with a senior librarian from the University of Warwick. To capture the relevant studies, we applied a search strategy that ensured correct use of Boolean operators, truncation, and medical subject headings (MeSH). The search strategy (Table 3) was based on keywords in five domains which were all combined using the “AND” operator: (i) overall topic of interest – antibiotic resistance, (ii) construct of interest – knowledge and attitudes, (iii) type of measurement – instruments, (iv) target population – healthcare professionals, nurse, doctor,

and (v) therapeutic area of interest- UTIs.

**Table 3. Search Strategy**

| #overall topic of interest | (Drug resistance, microbial OR drug resistance, multiple, bacterial OR antimicrobial resistance OR drug resistance, bacterial) OR ((antimicrobial" OR antibiotic* OR antibacterial*) AND (resistan* OR misuse OR overuse OR mis-use OR over-use OR inappropriate)) |
| --- | --- |
| #construct of interest | (Health Behaviour OR health knowledge, attitudes, practice, OR attitude to health) OR (attitude* OR perception* OR belief* OR opinion* OR thought* OR feeling* OR view or views or experience*) |
| #type of measurement | (Instrument* or tool OR tools OR survey* OR measure*OR psychometric) |
| #target population | (Healthcare professionals OR healthcare workers OR healthcare providers or physician or nurse or doctor or general practitioner* OR gp OR family doctor or primary care or primary healthcare or family practice or doctor* or healthcare professional or physician*) |
| #therapeutic area of interest | (Urinary tract infection* OR uti* OR tract infection* OR urinary infection* or bladder infection*) |

Where one or more psychometric property(ies) was assessed, the Consensus-based Standards for the selection of health Measurement Instruments (COSMIN) checklist (Mokkink et al., 2019) was used to evaluate the psychometric property(ies) of the included instrument. The COSMIN checklist is a tool designed for the assessment of both the risk of bias (Mokkink et al., 2019, Terwee et al., 2011) and the quality criteria for health instruments (Lorente et al., 2020). The risk of bias was not assessed, because the systematic review was non-interventional but instead only the quality criteria for measurement properties were used. Due to the lack of specific tools for appraisal of knowledge and attitude instruments, the COSMIN tool was considered appropriate for this review as it covers an even wider range of aspects than only those relevant for educational interventions (Mokkink et al., 2019). It has been widely used in the quality assessment of instruments (Whiting et al., 2017). The COSMIN tool provides separate checklists (referred to as boxes) for each type of measurement property, for example, box A is for internal consistency, box B for reliability and so forth. Boxes A-H are for different types of measurement properties and have their own associated quality questions on whether a study on a specific measurement property meets the standards for good instrument quality. See Mokkink et al. (2010) for a full explanation of the COSMIN checklist.

Reviewer #1: comment 4. Were the included studies were finalized after the risk biased assessment? Is any scale was used for the risk bias assessment and the protocols at all.

>>>Author Response to reviewer 1 comment 4. As noted above, no tool was used for the risk bias assessment, as the main aim of this study was not to understand the quality of studies that reported measurements of knowledge and attitudes but instead to evaluate the reported psychometric properties of included instruments. As stated in the previous comment response (comment 3), the review did not focus on any interventions and hence a risk of bias assessment was not required.

Reviewer #1 comment 5. The main evidence which has been drawn from the present review is not cleared. Very specific evidence must be mentioned that must represent the whole picture of the review. Such measuring scales and other factors must be controlled and the conclusion and the evidence from the review must be cleared that would be helpful for the readers and researchers in the field

>>>Author Response to reviewer 1 comment 5. We apologise that our original synthesis was not sufficiently clear. This comment is now addressed in the discussion, starting at line 453:

The findings reported in this review show that there are no instruments for which there is evidence of all the required psychometric properties of validity, reliability, acceptability, feasibility, and potential for educational impact to enable adequate measurement of healthcare professionals’ knowledge and attitudes towards the use of antibiotics for the treatment of UTIs. Measurement instruments with satisfactory psychometric properties are required to yield useable findings. Without such useable measures of knowledge and attitudes, it is impossible to determine if such knowledge and attitudes require improvement and, if so to plan and evaluate appropriate ameliorative interventions.

The findings reported in this review also support the need for solid theoretical foundations to define the constructs of knowledge and attitude from which such instruments need to be developed. Previous researchers of similar studies have reported a variation of constructs used for measurement instruments addressing inappropriate antibiotic use (Abubakar et al., 2021). Varying definitions of knowledge and attitudes exist (Teixeira Rodrigues et al., 2016, Michie et al., 2005) and some researchers fail to define the constructs measured (Morgado et al., 2017).

Reviewer #1 comment 6. Recent references are needed to be included in the introduction and discussion parts.

>>>Author Response to reviewer 1 comment 6. Thank you for suggesting that we update the introduction and discussion with recent references. This is now reflected across the introduction and discussion to reflect recent articles. For example, only references not more than 5 years have been used in the first paragraph, and interventional studies to further support the research rationale. The discussion has also been updated with recent research.
